# Neonatal Non-metabolic Seizure Distribution in Relation to Biochemical Abnormalities

**DOI:** 10.7759/cureus.79723

**Published:** 2025-02-26

**Authors:** Kothakapu Rajesh Reddy, Dnyaneshwar Potpalle, Abdul Muqeeth Mohammed, Mummareddi Dinesh Eshwar

**Affiliations:** 1 Paediatrics and Child Health, Mahavir Institute of Medical Sciences, Vikarabad, IND; 2 Biochemistry, Mahavir Institute of Medical Sciences, Vikarabad, IND; 3 General Medicine, Mahavir Institute of Medical Sciences, Vikarabad, IND

**Keywords:** biochemical investigation, emergency room, metabolic diseases, neonatal seizure, newborns, seizure

## Abstract

Introduction

Neonatal seizures are a significant neurological manifestation in the early days of life. They can cause long-term developmental disabilities and increase mortality rates if not properly managed. They can also be a sign of underlying neurological disorders, metabolic disturbances, or hypoxic-ischemic injury. Among the numerous etiologies, birth asphyxia, neonatal meningitis, and biochemical abnormalities are widespread. Biochemical abnormalities occur either as an underlying cause or as an associated abnormality. So biochemical anomalies should be excluded in every case of neonatal seizure, despite the presence of other causes of neonatal seizures. Early recognition and treatment of these biochemical disturbances are essential for optimal management and satisfactory outcomes. We assessed the proportion of newborn seizures caused by different etiologies in the current study with due emphasis on biochemical causes.

Methods

A prospective observational study was conducted in a university-affiliated teaching hospital between November 2015 and April 2017 (17 months). The study recruited 100 neonates who had a history of seizures. A comprehensive history and examination have been recorded upon admission, along with appropriate investigations. The study was approved by the institutional ethics committee, and all participants provided their voluntary informed consent.

Results

Among 100 cases of neonatal seizures, biochemical abnormalities were identified in 75 cases, comprising 52 (69.3%) non-metabolic seizures and 23 (30.6%) pure metabolic convulsions. The predominant biochemical abnormalities in neonatal seizures are hypoglycemia in 34 of 75 cases (45.3%) and hypocalcemia in 17 of 75 cases (22.7%).

The most frequent abnormality in non-metabolic seizure patients was low blood sugar in 23 (44.2%), followed by low calcium in 12 (23.1%), low phosphate in eight (15.4%), high sodium in six (11.5%), low sodium in two (3.8%), and low magnesium in one (1.9%). Out of the 23 subjects with hypoglycemia in non-metabolic seizures, 12 (52.1%) were associated with perinatal asphyxia, while 11 (47.9%) were linked to septicemia.

The biochemical abnormalities identified in metabolic seizures were hypophosphatemia in four (17.4%), hypomagnesemia in three (13%), hypoglycemia in 11 of 23 patients (47.8%), and hypocalcemia in five (21.7%).

Conclusion

Improving outcomes and quality of life for newborns impacted by non-metabolic neonatal seizures requires a comprehensive approach to treatment. This includes the appropriate therapy, the results of the biochemical testing, and the clinical evaluation.

## Introduction

The term "seizure" describes a periodic, abnormal, uncontrolled brain electrical activity. A number of symptoms, such as atypical movement or behavior, sensory disruption, autonomic dysfunction, impairment, or loss of consciousness, may indicate this illness [1,2]. Seizures typically manifest in infants within the first 10 days of their lives. For a full-term newborn, seizures must manifest within the first four weeks of life, whereas for a preterm infant, they must occur no later than 44 weeks post-conception [3]. 

An estimated 0.95-3.5 per 1000 live births is affected by neonatal seizures, a prevalent neurological disorder [4]. During this phase of life, seizure frequency is significantly higher than at other times of life. Seizures occur in just 2.8 out of 1000 infants weighing 2600-3999 g [5-7]. However, in babies under 1500 g, the seizure incidence might reach 57.5 per 1000. 

Neonatal seizures constitute one of the most prevalent neurological crises in neonates. These illnesses might be linked to a broad range of underlying disorders, from metabolic problems to structural brain abnormalities. Having seizures in a baby is the most glaring indicator of a neurological disorder at this critical period [8]. 

One of the most crucial aspects is the diagnosis of neonatal seizures; the next is determining their origin and finally administering therapy. To start, specialized treatment may be required for seizure disorders, which often occur alongside other serious health issues. Secondly, the critical supporting measures needed for the treatment of related illnesses may be obstructed by newborn convulsions. A few examples of these treatments include nutrition and aided breathing. Finally, experimental data suggests that seizures can potentially induce brain injury [9]. 

Seizures can present in a variety of forms; some of these are generalized tonic-clonic seizures, multifocal-clonic seizures, and subtle seizures. Timely diagnosis and treatment are essential to prevent brain injury, as delays can result in significant problems [10]. Seizures, while not a conclusive diagnosis, indicate a more severe medical illness affecting the central nervous system, often resulting from metabolic or systemic issues. 

Some of the most common reasons why babies have seizures are birth defects, biochemical abnormalities, and neonatal meningitis. Birth asphyxia and metabolic abnormalities are two more possible causes. An underlying cause or an associated disorder could be shown by biochemical abnormalities. These two results are not out of the question. If optimal therapy and desired outcomes are to be achieved, it is imperative to promptly identify and address these metabolic anomalies. The potential for metabolic abnormalities to produce newborn seizures should thus be considered even when other reasons, such as meningitis, hypoxia, or anatomical abnormalities, are also present. 

This study aimed to detect metabolic abnormalities related to seizures in newborns. The goal in doing this would be to improve the prognosis and allow for earlier treatment. This current work is designed to find out how biochemical abnormalities relate to the prevalence of non-metabolic seizures.

## Materials and methods

The study was conducted at JJM Medical College located in Davangere, India, after obtaining approval from the institute's Institutional Ethics Committee (approval number: JJMMC/IEC/Sy-44/2015). One hundred infants diagnosed with seizures from November 2015 to April 2017, who met the inclusion criteria, had been enrolled in the investigation following the submission of written informed consent by their parents or legal guardians. Eligibility for inclusion is contingent upon the newborn being less than 28 days old. Regardless of gestational age or birth weight, the research includes neonates with seizures who exhibit at least one of the following clinical types of seizures: subtle seizures, multifocal-clonic seizures, focal-clonic seizures, myoclonic seizures, or generalized tonic seizures. In contrast, neonates with suspicious seizures, tremors, and tetanic spasms, as well as those with major congenital malformations, were excluded from the study.

A comprehensive prenatal, natal, and postnatal history was recorded according to the included pro forma. Fundamental data regarding the convulsing neonate were noted. We conducted a thorough physical examination of the convulsing neonate. Clinical particulars of each seizure event have been recorded. Relevant investigations were carried out based on the clinical presentation. Testing will include a full blood count as well as sepsis screening measures (such as total leukocyte count (TLC), absolute neutrophil count (ANC), immature-to-total neutrophil ratio, and C-reactive protein (CRP)), blood glucose, and serum electrolytes that involve potassium, phosphorus, sodium, calcium, and magnesium. If metabolic illnesses are suspected, further metabolic screening approaches include checking serum lactate, urine for reducing substances, urine ketones, and serum ammonia. Lumbar puncture was considered in all cases when meningitis or septicemia was suspected. All neonates who had seizures had a neurosonogram to make sure there wasn't any intraventricular or parenchymal hemorrhage or congenital brain abnormalities. An electroencephalogram (EEG) was performed on every infant who required anticonvulsant medication. We conducted CT scans and MRIs of the brain as necessary.

Statistical analysis

We counted frequencies and percentages for categorical data and calculated basic statistics like standard deviation and average for continuous data. We looked at the correlation between the two variables using the chi-squared test. For this purpose, we compared the means of different numerical data sets using ANOVA. Outcomes had been considered significant when p<0.05.

## Results

The biochemical abnormalities found in 75 cases of neonatal seizures are enumerated in Table 1. Low blood sugar in 34 patients (45.3%) and low calcium levels in 17 patients (22.7%) were the most prevalent biochemical abnormalities found in neonatal seizures. Metabolic seizures were attributed to 23 (30.6%), and the remaining 52 (69.3%) were non-metabolic seizures. The most common biochemical abnormality in metabolic seizures is hypoglycemia found in 11 (47.8%) followed by hypocalcemia in five (21.7%), hypophosphatemia in four (17.4%), and hypomagnesemia in three (13%). Among non-metabolic seizures, the most common anomaly noted was hypoglycemia in 23 (44.2%) following which 12 subjects (23.1%) showed hypocalcemia and hypophosphatemia was found in eight neonates (15.4%). Hyponatremia and hypernatremia were found in two (3.8%) and six (11.5%) newborns, respectively.

**Table 1:** Comparison of biochemical abnormalities between metabolic and non-metabolic seizures

Biochemical parameters	Metabolic seizures N (%)	Non-metabolic seizures N (%)	Total N (%)	P-value
Hyponatremia	0 (0)	2 (3.8)	2 (2.7)	0.340
Hypernatremia	0 (0)	6 (11.5)	6 (8)	0.089
Hypoglycemia	11 (47.8)	23 (44.2)	34 (45.3)	0.773
Hypophosphatemia	4 (17.4)	8 (15.4)	12 (16)	0.827
Hypomagnesemia	3 (13)	1 (1.9)	4 (5.3)	0.048
Hypocalcemia	5 (21.7)	12 (23.1)	17 (22.7)	0.898

Table 2 shows that the majority of preterm neonates with seizures included in the study had hypoglycemia, i.e., 13 (40.6%), following which eight (22.2%) had hypocalcemia and six (16.7%) had hypophosphatemia. Hypoglycemia was also the most prevalent metabolic problem in term infants with neonatal seizures.

**Table 2:** Neonatal seizures and their distribution across biochemical domains SGA: small for gestational age; AGA: appropriate for gestational age

Biochemical parameters	Preterm (%)	AGA (%)	SGA (%)
Hyponatremia	0 (0)	2 (4.1)	0 (0)
Hypernatremia	2 (5.6)	3 (6.1)	1 (7.1)
Hypoglycemia	13 (36.1)	16 (32.7)	5 (35.7)
Hypophosphatemia	6 (16.7)	3 (6.1)	3 (21.4)
Hypomagnesemia	3 (8.3)	1 (2)	0 (0)
Hypocalcemia	8 (22.2)	4 (8.2)	5 (35.7)
Total	32	29	14

The distribution of biochemical abnormalities by gender is depicted in Table 3. The most common biochemical aberration in both male and female children was found to be hypoglycemia.

**Table 3:** Distribution of overall biochemical abnormalities with gender

Biochemical parameters	Female (%)	Male (%)	P-value
Hyponatremia	1 (2.3)	1 (1.8)	0.017
Hypernatremia	6 (13.6)	0 (0)	0.001
Hypoglycemia	15 (34.1)	19 (33.9)	0.986
Hypophosphatemia	4 (9.1)	8 (14.3)	0.427
Hypomagnesemia	2 (4.5)	2 (3.6)	0.805
Hypocalcemia	7 (15.9)	10 (17.8)	0.409
Total	35 (46.7)	40 (53.3)	

Table 4 shows the distribution of non-metabolic seizures across a range of biochemical disorders. Hypoglycemia was found to be the most prevalent biochemical anomaly in both birth asphyxia and septicemia. However, the most common and the only biochemical anomaly in cases of intracranial bleed was hypocalcemia.

**Table 4:** Distribution of non-metabolic seizures with respect to biochemical abnormalities

Biochemical parameters	Birth asphyxia (%)	Intracranial bleed (%)	Septicemia (%)
Hyponatremia	2 (5.3)	0 (0)	0 (0)
Hypernatremia	0 (0)	0 (0)	6 (15.8)
Hypoglycemia	12 (31.6)	0 (0)	11 (28.9)
Hypophosphatemia	2 (5.3)	0 (0)	6 (15.8)
Hypomagnesemia	1 (2.6)	0 (0)	0 (0)
Hypocalcemia	4 (10.5)	1 (33.3)	7 (18.4)
Total	21	1	30

The importance of the correlation between etiology and gestational age is seen in Table 5. This study found that metabolic abnormalities were responsible for 18 of the 38 cases of birth asphyxia that were evaluated. Investigational results showed that 11 cases were associated with hypoglycemia, five with hypocalcemia, and two with hyponatremia. Out of 38 cases of septicemia, 22 cases were associated with biochemical abnormalities, hypoglycemia was detected in 11 cases and hypernatremia in six cases, and five cases were related to hypocalcemia. One case of intracranial bleed was associated with hypocalcemia. 

**Table 5:** Association between etiology and gestational age (n=100) Chi-squared test; p=0.052; not significant SGA: small for gestational age; AGA: appropriate for gestational age

Biochemical parameters	Preterm (%)	AGA (%)	SGA (%)
Birth asphyxia/hypocalcemia	0 (0)	3 (6.1)	2 (13.3)
Birth asphyxia/hypoglycemia	2 (5.6)	8 (16.3)	1 (6.7)
Birth asphyxia/hyponatremia	0 (0)	2 (4.1)	0 (0)
Birth asphyxia (not associated with metabolic abnormalities)	2 (5.6)	16 (32.7)	2 (13.3)
Hypocalcemia	3 (8.3)	1 (2)	1 (6.7)
Hypoglycemia	6 (16.7)	2 (4.1)	3 (20)
Hypocalcemia+hypomagnesemia	2 (5.6)	0 (0)	0 (0)
Hypomagnesemia	1 (2.8)	0 (0)	0 (0)
Intracranial bleed	1 (2.8)	0 (0)	0 (0)
Septicemia (not associated with metabolic abnormalities)	8 (22.2)	6 (12.2)	2 (13.3)
Septicemia/hypernatremia	2 (5.6)	3 (6.1)	1 (6.7)
Septicemia/hypocalcemia	3 (8.3)	0 (0)	2 (13.3)
Septicemia/hypoglycemia	5 (13.9)	5 (10.2)	1 (6.7)

## Discussion

In the present study, neonatal seizures were predominantly attributed to septicemia, followed by birth asphyxia, pure metabolic causes, and cerebral hemorrhage, highlighting the significant role of infections and perinatal complications in the etiology of neonatal seizures. Biochemical abnormalities were observed in 75 out of 100 newborns with neonatal seizures, while the remaining 25 exhibited no biochemical abnormalities, indicating that a portion of neonatal seizures may occur without any detectable biochemical disturbance. According to the present study, hypoglycemia, the most common biochemical abnormality, was identified in 23 newborns with non-metabolic seizures and 11 neonates with metabolic seizures. The severity of encephalopathy and cellular damage were shown to be correlated with the severity of hypoglycemia, according to the results presented by Lanska and Lanska [11].

Table 6 shows the causes of neonatal seizures across various studies.

**Table 6:** Comparison of biochemical abnormalities in metabolic and non-metabolic seizures across different studies

Biochemical abnormalities	Kumar et al. [12] (n=35)	Sood et al. [13] (n=59)	Madhusudhan et al. [14] (n=120)	Present study (n=100)
Metabolic seizure	10	20	23	23
Non-metabolic seizure	12	9	29	52
No abnormalities	13	30	68	25

Hypoglycemia was most commonly associated with both birth asphyxia and septicemia as mentioned in Table 4. Hypocalcemia was observed in birth asphyxia, intracranial bleed, and septicemia, with a notably higher percentage in intracranial bleed. Hypernatremia was predominantly seen in septicemia. Hypophosphatemia occurred in both birth asphyxia and septicemia. Hypomagnesemia was rare across all conditions. Hyponatremia was uncommon in both intracranial bleed and septicemia, but present in a small percentage of birth asphyxia cases. Hypoglycemia was found to be a frequent abnormality in birth asphyxia and septicemia, while hypocalcemia and hypernatremia were notable in septicemia. The presence of biochemical imbalances like these is important for the clinical management and treatment of neonatal seizures. The biochemical abnormalities associated with birth asphyxia observed in the present study, including hypoglycemia, hypocalcemia, hypomagnesemia, and hyponatremia, are consistent with the findings of the study conducted by Sood et al. [13].

Babies born prematurely are at elevated risk of hypoglycemia due to their reduced glycogen stores and insufficient early postnatal feeding. Hypoglycemic seizures were the predominant seizure type occurring within the initial three days. Brain damage can be serious if hypoglycemia is not diagnosed quickly. Study after study on hypoglycemia by Lilien and colleagues [15] found that small for gestational age (SGA) was the culprit in 41% of newborns' cases of low blood sugar. The prevalence of hypoglycemia, which can cause seizures in 30.2% of cases, is three times more in preterm neonates (12.6% compared to 3.6% in term babies), according to the research of Singhal et al. [16]. 

Table 7 elaborates on the various biochemical abnormalities in different studies.

**Table 7:** Comparison of electrolyte and metabolic abnormalities (including sodium, glucose, magnesium, calcium, and phosphate imbalances) observed in different studies and the present study

Biochemical parameters	Kumar et al. [12] (n=35)	Sood et al. [13] (n=59)	Madhusudhan et al. [14] (n=120)	Present study (n=100)
Hyponatremia	10	5	19	2
Hypoglycemia	11	14	17	34
Hypermagnesemia	1	0	0	0
Hypomagnesemia	3	5	1	2
Hypernatremia	0	0	3	6
Hypocalcemia+hypomagnesemia	0	0	1	2
Hypophosphatemia	0	0	0	12
Hyperphosphatemia	3	0	0	0
Hypocalcemia	7	14	11	17

After looking at 23 with pure metabolic abnormalities, we found that hypocalcemia was the underlying cause in five newborns. Recognizing hypocalcemia and administering intravenous calcium immediately is crucial. Neonatal blood calcium levels are lower than normal in infants fed with cow milk instead of breast milk, according to research by Cockburn et al. [17]. However, our babies were exclusively breastfed. Two individuals were found to have hypocalcemia, and one had hypomagnesemia to a particular degree throughout our research. Convulsive illness, which can cause permanent brain damage, can be a symptom of magnesium insufficiency. Intrauterine growth restriction (IUGR), temporary hypoparathyroidism, diabetic moms, and other medical issues are among the many potential causes of hypomagnesemia in newborns. Since the convulsive state is maintained by increasing the amount of magnesium that the kidneys excrete, a brief intravenous calcium infusion worsens hypomagnesemia. For those with hypocalcemia and hypomagnesemia, the situation can be reversed by increasing magnesium intake. Therefore, when given orally to infants, magnesium is likely to treat hypocalcemia as well as hypomagnesemia. One preterm newborn with neonatal convulsions was found to have a cerebral hemorrhage. The improved risk of intraventricular hemorrhage in preterm infants is associated with their smaller periventricular blood vessel support systems and more fragile blood vessel walls. 

## Conclusions

It is crucial to use a comprehensive strategy while identifying and treating non-metabolic neonatal seizures since this condition is complex and difficult. The distribution of these seizures in relation to metabolic diseases must be understood for the purpose of providing focused and effective therapy. By doing thorough clinical evaluations and biochemical testing and prescribing suitable medications, medical professionals can enhance the quality of life and outcomes for neonates impacted by this seizure disorder. 
